# Diagnostik und Therapie des Typ 1 Diabetes mellitus (Update 2026)

**DOI:** 10.1007/s00508-025-02628-1

**Published:** 2026-04-30

**Authors:** Monika Lechleitner, Susanne Kaser, Sabine Hofer, Friedrich Hoppichler, Michael Roden, Raimund Weitgasser, Lisa Fruehwald, Bernhard Ludvik, Astrid Feder, Yvonne Winhofer-Stöckl, Alexandra Kautzky-Willer, Guntram Schernthaner, Thomas C. Wascher, Martin Clodi

**Affiliations:** 1 Avomed – Arbeitskreis für Vorsorgemedizin und Gesundheitsförderung in Tirol, Innsbruck, Österreich; 2 Medizinische Universität Innsbruck, Department für Innere Medizin 1, 6020 Innsbruck, Österreich; 3 Department für Kinder- und Jugendheilkunde Pädiatrie I, Endokrinologie, Medizinische Universität Innsbruck, Innsbruck, Österreich; 4 Abteilung für Innere Medizin, Krankenhaus der Barmherzigen Brüder Salzburg, Salzburg, Österreich; 5 Klinik für Endokrinologie und Diabetologie, Medizinische Fakultät, Heinrich-Heine-Universität, Düsseldorf, Deutschland; 6 Institut für Klinische Diabetologie, Deutsches Diabetes-Zentrum (DDZ), Leibniz-Zentrum für Diabetesforschung, Düsseldorf, Deutschland; 7 Deutsches Zentrum für Diabetesforschung (DZD e. V.), München-Neuherberg, Deutschland; 8 Kompetenzzentrum Diabetes, Mavie Med Privatklinik Wehrle-Diakonissen, Salzburg, Österreich; 9 Universitätsklinik für Innere Medizin I, LKH Salzburg – Universitätsklinikum der Paracelsus Medizinischen Privatuniversität, Salzburg, Österreich; 10 5. Medizinische Abteilung für Endokrinologie, Rheumatologie und Akutgeriatrie, Wilhelminenspital der Stadt Wien, Wien, Österreich; 11 1. Medizinische Abteilung mit Diabetologie, Endokrinologie und Nephrologie und Karl Landsteiner Institut für Adipositas und Stoffwechselerkrankungen, Klinik Landstrasse, Wien, Österreich; 12 Klinische Abteilung für Endokrinologie und Stoffwechsel, Universitätsklinik für Innere Medizin III, Medizinische Universität Wien, Wien, Österreich; 13 Gender Medicine Unit, Klinische Abteilung für Endokrinologie und Stoffwechsel, Universitätsklinik für Innere Medizin III, Medizinische Universität Wien, Wien, Österreich; 14 1. Medizinische Abteilung mit Diabetologie, Endokrinologie und Department für Nephrologie, Krankenanstalt Rudolfstiftung, Wien, Österreich; 15 1. Medizinische Abteilung, Hanusch-Krankenhaus, Wien, Österreich; 16 Johannes Kepler Universität Linz, ICMR – Institute for Cardiovascular and Metabolic Research, Linz, Österreich; 17 Abteilung für Innere Medizin, Konventhospital der Barmherzigen Brüder Linz, Linz, Österreich

**Keywords:** Stadieneinteilung, Autoimmunerkrankungen, Insulintherapie, Staging, Autoimmune diseases, Insulin therapy

## Abstract

Die Leitlinie nimmt Bezug auf die Diagnostik einschließlich begleitender Autoimmunerkrankungen bei Typ-1-Diabetes mellitus, die Insulintherapie und die glykämischen Zielwerte.

## Abstract (en)

The guidelines summarize the diagnostics of type 1 diabetes mellitus, including accompanying autoimmune diseases, insulin therapy regimens and glycemic target values.

In Bezug auf die Definition werden der Typ-1- und Typ-2-Diabetes mellitus als heterogene Erkrankungen mit unterschiedlicher klinischer Präsentation dargestellt, wobei die Klassifizierung in Typ-1- und Typ-2-Diabetes für die Therapieentscheidung von grundlegender Bedeutung ist [1–3]. Der Typ-1-Diabetes mellitus betrifft rund 5–10 % aller Diabeteserkrankungen. Ein Großteil der Neumanifestationen eines Typ-1-Diabetes tritt im Kindes- und Jugendalter auf [1, 3, 4].

Entsprechend der Auswertungen der Daten aus 201 Ländern wurde für das Jahr 2021 geschätzt, dass weltweit 8,4 Mio. Menschen einen Typ-1-Diabetes aufweisen, davon sind 18 % jünger als 20 Jahre, 64 % zwischen 20 und 50 Jahren und 19 % über 60 Jahre alt [5]. Bis zum Jahr 2040 wird mit einem Anstieg der Prävalenz von Typ-1-Diabetes auf 13,5–17,4 % aller Diabetesfälle gerechnet [5].

Der Entwicklung des Typ-1-Diabetes liegt eine zellulär mediierte Autoimmundestruktion der pankreatischen Beta-Zelle zugrunde [1, 2]. Der Typ-1-Diabetes wird durch eine fehlende oder inadäquat niedrige Insulinsekretion gemessen mittels C‑Peptid und Vorliegen von einem oder mehreren Beta-Zell-spezifischen Autoantikörpern definiert, wobei bei Manifestation im Erwachsenenalter bei 5–10 % aller Betroffenen kein Autoantikörper nachweisbar ist, bei diesen Patienten wird die Diagnose dann anhand der Klinik zusammen mit einem C‑Peptid < 200 pmol/l (< 0,2 ng/ml) gestellt (Abb. 1 und 2).

## Stadieneinteilung des Typ 1 Diabetes

Bei der Einteilung von Typ-1-Diabetes in Stadien werden der Nachweis von Beta-Zell-spezifischen Antikörpern und das Maß an Dysglykämie berücksichtigt.

**Abb. 1:**
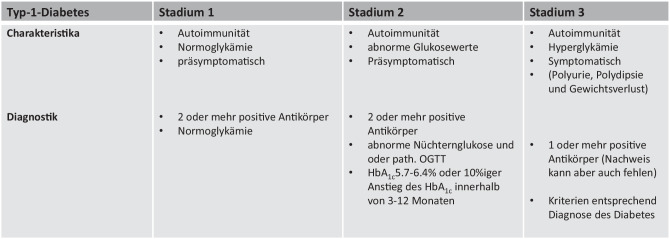
Stadieneinteilung des Typ 1 Diabetes mellitus (nach ADA 2025) [1]

Menschen, bei denen ein positiver Antikörper detektiert wird, werden als „at risk“ für Diabetes klassifiziert. Sind 2 oder mehrere Beta-Zell-spezifische Autoantikörper nachweisbar, werden Individuen als Typ-1-Diabetes Stadium 1 klassifiziert. Diese Individuen haben keine Diabetes-spezifischen Symptome und keine Dysglykämie. Typ-1-Diabetes Stadium 2 ist gekennzeichnet durch das Vorliegen von 2 oder mehr positiven Antikörpern, Dysglykämie, jedoch ohne klinische Zeichen einer Hyperglykämie (Polyurie, Polydipsie, Gewichtsverlust etc.). Typ-1-Diabetes Stadium 3 ist gekennzeichnet von positiven Autoantikörperbefunden und dem Vorliegen einer Hyperglykämie, wobei diese die ADA-Schwellenwerte (HbA_1c_ ≥ 6,5 %/≥ 48 mmol/mol oder Nüchternglukose ≥ 126 mg/dl oder 2-h-Wert im oGTT ≥ 200 mg/dl) erreicht. Der langjährige Verlauf von Typ-1-Diabetes wird als Stadium 4 bezeichnet (Abb. 1; [1, 6]).

Die diagnostischen Autoimmunmarker inkludieren GAD65-Antikörper, Insulin-Antikörper, Antikörper gegenüber Tyrosinphosphatase IA‑2 und IA-2beta sowie Antikörper gegenüber Zinktransporter 8 (ZnT8) [1, 4, 6–8]. GAD65, Insulin-Antikörper, IA‑2 und ZnT8 gelten als jene Antikörper, die für das Screening auf Typ-1-Diabetes verwendet werden sollten. Derzeit wird die Diagnose eines immunologisch vermittelten Typ-1-Diabetes meist im hyperglykämischen Stadium 3 gestellt, und bei 85–90 % der Patienten findet sich zumindest ein positiver Antikörperbefund ([1, 2]; Abb. 1).

Bei Diagnosestellung in früheren Stadien, insbesondere in den Stadien 1 und 2, sind strukturierte Folgeuntersuchungen notwendig. Die Intensität der Folgeuntersuchung hängt vom Alter ab. Bei jüngeren Kindern sind eine raschere Progredienz der Beta-Zell-Destruktion und raschere Insulinpflichtigkeit zu erwarten. Die Kontrolluntersuchungen von metabolischen Parametern (HbA_1c_, Glukosewerte, eventuell CGM-Messungen) sind deshalb in Altersgruppen gestaffelt, je jünger die Individuen, desto höher die Intensität der Kontrolluntersuchungen.

Für eine genetische Prädisposition spricht die starke Assoziation zwischen Typ-1-Diabetes und dem HLA-Genotypus, insbesondere DQA und DQB [1, 2].

Das Risiko zur Entwicklung eines Typ-1-Diabetes ist bei Verwandten von Menschen mit Typ-1-Diabetes um das 15- bis 20-Fache erhöht und beträgt 6–7 % bei Geschwisterkindern und 25–50 % bei eineiigen Zwillingen [9–11]. Bei Angehörigen von Menschen mit Typ-1-Diabetes wurde im Rahmen von Studien das Risiko einer Diabetesmanifestation bei positiven Antikörperbefunden untersucht. Aus einer multinationalen Studie bei Kindern geht hervor, dass bei mehr als 2 positiven Antikörpern rund 70 % innerhalb der nächsten 10 Jahre und 84 % innerhalb von 15 Jahren einen Typ-1-Diabetes entwickeln [12].

Die Ausprägung der klinischen Symptome eines manifesten Typ-1-Diabetes ist variabel mit Polyurie, Polydipsie, Schwächegefühl, Sehstörungen, Infektneigung und Gewichtsverlust als typischen Anzeichen einer metabolischen Entgleisung bis hin zur diabetischen Ketoazidose. Die diabetische Ketoazidose und begleitende gastrointestinale Beschwerden können v. a. bei Kindern im Rahmen der Erstmanifestation eines Typ-1-Diabetes beobachtet werden [1, 11, 13].

Neben den klassischen Autoimmunformen des Typ-1-Diabetes wurden in den letzten Jahren – insbesondere in afrikanischen und asiatischen Ethnien – idiopathische Varianten eines Typ-1-Diabetes mellitus beschrieben. Dabei zeigte sich kein serologischer Hinweis auf eine Beta-Zell-Autoimmunität, jedoch ein permanenter Insulinmangel mit Ketoazidoseneigung [1, 14].

Differenzialdiagnostisch ist eine rasche Entwicklung einer ausgeprägten Insulindefizienz unter Therapie mit Checkpointinhibitoren möglich [1, 2, 15] (s. Kapitel medikamentös induzierte Diabetesformen).

**Abb. 2:**
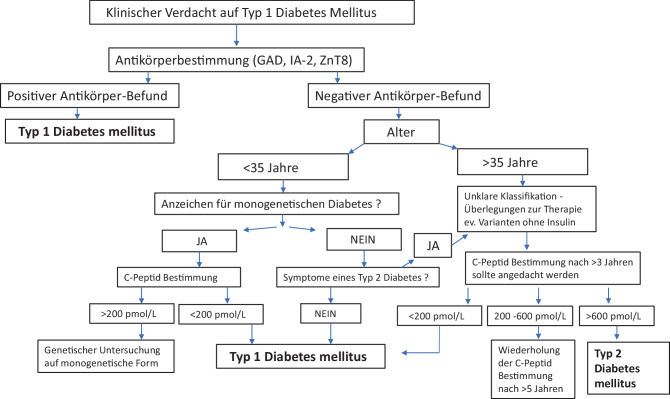
Flowchart zur Diagnostik bei Verdachtsdiagnose eines Typ 1 Diabetes bei Erwachsenen (nach ADA 2025) [1]

**Abb. 3:**
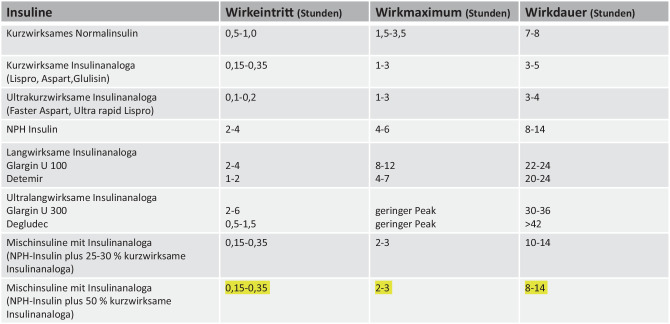
Insulinpräparate und Charakteristika bei subkutaner Gabe (nach [1, 8])

Die vormals als „latent autoimmune diabetes in adults“ (LADA) bezeichnete Diabetesvariante stellt ein phänotypisches Mischbild dar, das von einem Autoantikörper-positiven Typ-1-Diabetes mit frühzeitiger Insulindefizienz bis zu Formen mit vorübergehend ausreichender Beta-Zell-Reserve und Symptomen des metabolischen Syndroms reicht (Abb. 2; [3, 6, 16–18]).

## Therapie des Typ 1 Diabetes mellitus

Eine Grundlage zur erfolgreichen Umsetzung der umfassenden Therapie (Insulintherapie, Glukosekontrolle, Lebensstilmaßnahmen, präventive Maßnahmen betreffend diabetischer Akut- und Spätkomplikationen) ist die Teilnahme an einer strukturierten Schulung und damit Übernahme der Entscheidungskompetenz in der Therapieumsetzung durch die Betroffenen selbst (Beachtung der Bedeutung der Partizipation der Menschen mit Diabetes, s. Leitlinie „Diabetesschulung und -beratung bei Erwachsenen mit Diabetes“ sowie „Diabetes im Kindes- und Jugendalter“).

Die Insulintherapie stellt bei Typ-1-Diabetes mellitus eine lebensnotwendige Hormonersatztherapie dar. Die umfassende interprofessionelle Betreuung der Menschen mit Typ-1-Diabetes sollte grundsätzlich an einem diabetologischen Zentrum bzw. bei einer Ärztin/einem Arzt mit entsprechender Schwerpunktausbildung für Diabetologie/Endokrinologie erfolgen, insbesondere auch im Hinblick auf das Screening auf diabetische Spätkomplikationen und Komorbiditäten (s. Leitlinien „Diabetische Nierenerkrankung“, „Diabetische Augenerkrankung“, „Diabetische Neuropathie und diabetischer Fuß“, „Koronare Herzerkrankung und Herzinsuffizienz“) [1, 2, 19, 20].

## Insulintherapie bei Diabetes mellitus Typ 1

### Insuline

Zur Insulintherapie werden in Österreich Insulinanaloga in einer Konzentration von 100 IE pro ml (U 100) verwendet. Insuline mit höheren Konzentrationen sind Insulin glargin U 300 sowie für Patienten mit besonders hohem Insulinbedarf Insulin lispro U 200 und Humulin R U 500. Diese höher konzentrierten Insuline stehen nur in Form von Fertigpens zur Verfügung, um das Risiko von Verwechslungen bei Ampullenwechsel und damit Überdosierungen zu reduzieren.

Die routinemäßige Verabreichung von Insulin erfolgt subkutan mittels Pen oder durch eine Insulinpumpe und automatisierte Insulinabgabe(AID)-Systeme. Stoffwechselentgleisungen oder Komorbiditäten (perioperative Versorgung) können kurzfristig eine intravenöse Verabreichung von kurz wirksamen Insulinen erforderlich machen. Insuline stehen als kurz wirksame, lang wirksame Insuline und Insulinanaloga sowie als Mischinsuline zur klinischen Anwendung zur Verfügung (Abb. 3).

*Kurz wirksame Insulinanaloga *(lispro/ultra rapid lispro, Aspart/Faster-Aspart, glulisin) werden seit rund 3 Jahrzehnten in der Diabetestherapie eingesetzt [19]. Der gegenüber Normalinsulin raschere Wirkeintritt und die geringere Wirkdauer der kurz wirksamen Insulinanaloga haben die Lebensqualität der Menschen mit Diabetes insofern verbessert, als kein bzw. ein geringerer Spritz-Ess-Abstand mehr eingehalten werden muss. In klinischen Studien war die Hypoglykämierate, insbesondere für schwere und nächtliche Hypoglykämien, unter kurz wirksamen Insulinanaloga deutlich geringer als unter Normalinsulin [21–24].

Eine weitere Entwicklung stellen *ultrakurz wirksame Insulinanaloga* dar, wobei durch chemische Zusatzsubstanzen der Wirkeintritt von Insulin beschleunigt wird. Faster-Aspart (Zusatz von Niacinamid und L‑Arginin zu Insulin aspart) ist ca. 10 min rascher in der Zirkulation als Insulin aspart und zeigt eine 74 % höhere Insulinwirkung in den ersten 30 min nach Injektion [25]. Klinische Studien beschreiben für Menschen mit Typ-1- [26] und Typ-2-Diabetes [27] eine stärkere Reduktion des postprandialen Glukosespitzenwertes unter Faster-Aspart im Vergleich zu Insulin aspart. Ebenso zeigt Ultra rapid lispro (Zugabe von Citrat und Treprostinil) einen um ca. 10 min rascheren Wirkeintritt als Insulin lispro und eine vorteilhafte Beeinflussung der postprandialen Hyperglykämie [28, 29].

Die Entwicklung *lang wirksamer Insulinanaloga* (Insulin glargin U 100, Insulin detemir) hatte zum Ziel, eine gegenüber NPH-Insulin flachere Wirkkurve und längere Wirkdauer zu erzielen. Lang wirksame Insulinanaloga zeigten in klinischen Studien gegenüber NPH-Insulin eine Reduktion v. a. nächtlicher Hypoglykämien [30–32]. Von Vorteil in der klinischen Praxis und Handhabung ist auch das Vorliegen der lang wirksamen Insulinanaloga in Form einer klaren Lösung, während bei Applikation von NPH-Insulin eine vorausgehende Suspension des Insulins erforderlich ist.

Zu den sog. *ultralang wirksamen Insulinanaloga* zählen Insulin glargin U 300 und Insulin degludec [33, 34]. Die lange Wirkdauer und flache Wirkkurve der ultralang wirksamen Insulinanaloga ermöglicht eine Reduktion der Injektionshäufigkeit des basalen Insulins – üblicherweise auf 1‑mal täglich – und größere Flexibilität in der Wahl des Injektionszeitpunktes. In klinischen Studien wurden für die ultralang wirksamen Insuline insgesamt eine gegenüber Insulin glargin U 100 geringere Hypoglykämierate und geringere Variabilität der Blutzuckerschwankungen beschrieben. Für beide ultralang wirksamen Insulinpräparate liegen Studienreihen zum Einsatz bei Menschen mit Typ-1- und Typ-2-Diabetes mellitus vor [35–38].

Für das 1‑mal wöchentlich zu verabreichende Insulin icodec wurde gegenüber Insulin degludec bei Menschen mit Typ-1-Diabetes eine vergleichbare Effizienz in der HbA_1c_-Reduktion bei einer allerdings erhöhten Rate an klinisch signifikanten und nächtlichen Hypoglykämien beschrieben [40]. Auch für das ebenfalls 1‑mal wöchentlich zu verabreichende Insulin efistora alpha fanden sich gegenüber Insulin degludec eine vergleichbare HbA_1c_-Reduktion und aber höhere Hypoglykämierate [40, 41].

In Bezug auf die Sicherheit der Insulinanaloga konnten Metaanalysen unter Einschluss großer Patientenpopulationen aus Diabetesregistern keine Zunahme des Tumorrisikos für Insulinanaloga erheben [43].

Zur Verfügung stehen auch Biosimilars für Insulin glargin und Insulin lispro [44].

## Formen der Insulintherapie

Die *funktionelle Insulintherapie (Synonym: Basis-Bolus-Insulintherapie*; FIT/BBIT) mit 1‑ bzw. 2‑mal täglicher Verabreichung eines (ultra)lang wirksamen Insulinanalogons und eines (ultra)kurz wirksamen Insulinanalogons prandial bzw. als Korrekturinsulin ist – seit den Publikationen der DCCT-Studie – die Grundlage der Insulinbehandlung bei Typ-1-Diabetes [19, 45–48]. Im Rahmen der funktionell intensivierten Insulintherapie erfolgen multiple tägliche Insulininjektionen. Zur Anpassung der Insulintherapie und Übertragung der therapeutischen Entscheidungskompetenz an die betroffenen Menschen mit Typ-1-Diabetes ist dabei die Selbstkontrolle der Glukosewerte eine grundlegende Voraussetzung (s. Leitlinie „Blutzuckerselbstkontrolle“) sowie für den Konsum kohlenhydrathaltiger Nahrungsmittel die Kenntnis über die Einschätzung der Menge und Berechnung der entsprechenden Insulindosierung (s. Leitlinie „Ernährungsempfehlungen für Menschen mit Diabetes“). Kapilläre Messungen der Ketonkörperkonzentration unterstützen die Diagnostik bei einem erhöhten Risiko zur Entwicklung einer Ketoazidose [49].

Allen Menschen mit Typ-1-Diabetes sollte eine *Therapie mit einer Kombination aus Insulinpumpe und CGM mit automatischer Insulinabgabe* („Automated insulin delivery“[AID]-System) (im Sinn eines „hybrid closed-loop“) angeboten werden (s. Leitlinie „Diabetestechnologie“) [19].

Eine *konventionelle Form der Insulintherapie* (s. Leitlinie „Insulintherapie bei Typ 2 Diabetes mellitus“) sollte bei Menschen mit Typ-1-Diabetes nur noch in Ausnahmefällen zum Einsatz kommen.

Die funktionelle Insulintherapie stellt eine dem physiologischen Insulinsekretionsmuster angepasste Form der Insulinsubstitution dar [2, 19]. Bei normaler Beta-Zell-Funktion erfolgt eine basale Insulinsekretion im Fastenzustand kontinuierlich mit ca. 1,0 IE/h und diskontinuierlich entsprechend der Nahrungszufuhr. Die prandiale Freisetzung von Insulin beträgt bei Stoffwechselgesunden für Kohlenhydrate etwa 1,5 IE/10 g [50].

Generell liegt der Insulintagesbedarf bei normalgewichtigen Erwachsenen mit neu diagnostiziertem Typ-1-Diabetes bei insgesamt rund 0,4–1 IE/kg Körpergewicht [18]. Bei gewichtserhaltender Ernährung beträgt der *Anteil des basalen Insulins* 30–50 % der Gesamtdosis. Das basale Insulin ist von entscheidender Bedeutung für die Aufrechterhaltung eines normalen Stoffwechsels im Fastenzustand.

Bei der individuellen Anpassung der Insulindosis ist zu berücksichtigen, dass der absolute Insulinbedarf auch von der jeweiligen Insulinsensitivität der Menschen mit Typ-1-Diabetes abhängig ist. Die von Stoffwechselgesunden abgeleiteten Richtwerte für die Insulindosierung gelten nur für den Fall eines absoluten Insulinmangels und einer normalen Insulinsensitivität. Bei einem nur teilweisen Beta-Zell-Verlust reduziert die verbliebene Insulinrestsekretionsrate den täglichen Insulinbedarf bei Menschen mit Typ-1-Diabetes, während bei Insulinresistenz der Insulinbedarf erhöht ist. Für einen Großteil der Betroffenen muss die Insulindosierung deshalb individuell angepasst werden, v. a. unter Berücksichtigung des Ausmaßes des Insulindefizits, der Insulinsensitivität, der Pharmakokinetik und Pharmakodynamik der Insulinpräparate, der aktuellen Nahrungszufuhr und körperlichen Aktivität, sowie an hormonelle Veränderungen.

Die *Dosierung des prandialen Insulins* ergibt sich aus der Menge der zugeführten Kohlenhydrate (Kohlenhydrateinheiten = KE), tageszeitlichen Schwankungen (am Morgen höhere Dosis) sowie der Anpassung an die Glukosezielwerte (Korrekturinsulin). Die Dosis des prandialen Insulins für die zugeführte Menge an Kohlenhydraten beträgt bei Erwachsenen im Durchschnitt 1,0–2,0 IE/KE (1 KE entspricht einer Kohlenhydratmenge von 10 g). Für die Zufuhr von Protein bzw. Fett ist der Insulinbedarf wesentlich niedriger und wird in der täglichen Praxis zur Berechnung der Insulindosierung weniger häufig einbezogen [2, 8, 19]. Als eine Variante gilt dabei die Berechnung nach Pankowska [8, 51].

Unter Bezugnahme auf die Glukosewerte erfolgen Korrekturen des prandialen Insulins im Tagesverlauf beim Erwachsenen entsprechend der Grundregel, dass 1 IE kurz wirksames Insulin die Blutglukose um 30–40 mg/dl senkt. Dementsprechend erhöhen 10 g Kohlenhydrate die Blutglukose um ca. 30–40 mg/dl. Eine Anpassung der Insulindosis an den aktuellen Insulinbedarf ist stets erforderlich (z. B. bei Sportausübung, Infekten, Dehydratation) [2, 8, 19].

Insgesamt angestrebt wird unter Berücksichtigung der individuellen Gegebenheiten und v. a. des Hypoglykämierisikos eine normnahe, d. h. den Werten von Menschen ohne Diabetes angenäherte, Kontrolle der Glukosewerte [2, 3, 45].

## Glukosezielwerte

Bei Menschen mit Typ-1-Diabetes, die CGM als Standard of Care ablehnen, gelten weiterhin Blutglukosezielwerte im Rahmen der Selbstkontrollen: nüchtern bzw. vor den Mahlzeiten 80–110 mg/dl und vor dem Schlafengehen 110–130 mg/dl [2, 3, 45]. Ideale postprandiale Glukosespitzenwerte (Bestimmung 1–2 h nach Einnahme einer Mahlzeit) liegen unter 180 mg/dl [1]. Diese Glukosewerte entsprechen einem HbA_1c_-Wert von < 7,0 % [1–3, 45]. Nächtliche Glukosekontrollen (ca. 2.00–4.00 Uhr) werden bei Verdacht oder bei bekannter Neigung zu nächtlichen Hypoglykämien empfohlen und sollten darüber hinaus regelmäßig, je nach Stabilität der Stoffwechselkontrolle, alle 4 bis 8 Wochen vorgenommen werden.

Für die kontinuierlichen Glukosemessungen mittels subkutaner Glukosesensoren findet die Zeit im Zielbereich („time in range“, meist definiert durch Werte zwischen 70–180 mg/dl), als Indikator für die Qualität der Glukosekontrolle Anwendung [2, 45]. Bei einem Anteil von rund 70 % dieser Messungen im Zielkorridorbereich von 70–180 mg/dl ist ein Erreichen eines HbA_1c_-Wertes von unter 7 % (ideal HbA_1c_-Zielwert < 6,5 %) zu erwarten.

Grundlegend für die Zielwertdefinition und Wahl der Therapieform sind die Ergebnisse der DCCT/EDIC-Studie, die bereits 1993 aufzeigen konnte, dass bei Menschen mit Typ-1-Diabetes mit der Senkung des HbA_1c_-Wertes in die Nähe des Normbereichs das Risiko für mikroangiopathische Komplikationen signifikant reduziert wird [46–48]. In der ursprünglichen DCCT-Studie wurde die funktionelle Insulintherapie mit NPH-Insulin und Normalinsulin und auch ohne die heute bei Menschen mit Typ-1-Diabetes übliche kontinuierliche subkutane Glukosemessung umgesetzt. Die striktere glykämische Kontrolle war dabei mit einem erhöhten Hypoglykämierisiko assoziiert [46]. Für die in den Folgejahren entwickelten kurz und lang wirksamen Insulinanaloga konnte ein gegenüber NPH- bzw. Normalinsulin reduziertes Hypoglykämierisiko erhoben werden [21, 31, 32].

Die große klinische Bedeutung der Hypoglykämie geht auch aus Auswertungen der EURODIAB IDDM-Studie hervor, die nachweisen konnte, dass schwere Hypoglykämien mit Fremdhilfe bei Typ-1-Diabetes zu einer Verlängerung der QTc-Zeit und damit zu einem erhöhten Risiko für Herzrhythmusstörungen führen [52].

## Typ-1-Diabetes und Adipositas

Bisher wurden Menschen mit Typ-1-Diabetes als schlanke, insulinsensitive Individuen, bei denen der absolute Insulinmangel als Ursache der Hyperglykämie im Vordergrund steht, charakterisiert [1, 2]. Im Zuge des Anstieges der Prävalenz von Übergewicht und Adipositas finden sich auch häufiger übergewichtige und adipöse Menschen mit Typ-1-Diabetes bei Erstdiagnose, weshalb Adipositas kein Ausschlussgrund für die Autoantikörpertestung darstellen sollte [1, 53]. Rezente Untersuchungen zeigen auf, dass sowohl makro- als auch mikrovaskuläre Komplikationen bei Menschen mit Typ-1-Diabetes und klinischen Symptomen eines metabolischen Syndroms (von einigen Autoren auch als *Double Diabetes* bezeichnet) signifikant häufiger auftreten [53, 54].

Ob Substanzklassen, die in der Therapie des Typ-2-Diabetes zum Einsatz kommen, auch für Menschen mit Typ-1-Diabetes von Vorteil sein könnten, wurde in einer Reihe von klinischen Studien untersucht [55–58]. Anzuführen ist die Problematik eines derzeit fehlenden Zulassungsstatus als Zusatztherapie zu Insulin bei Menschen mit Typ-1-Diabetes.

Für Metformin wurden in klinischen Studien bei der Behandlung von Menschen mit Typ-1-Diabetes eine leichte Gewichtsreduktion, eine Reduktion des Insulinbedarfs, günstige Lipideffekte und Verbesserungen des HbA_1c_ beschrieben [8, 19, 59]. Die Studienresultate waren jedoch nicht konsistent und zeigten eine Abhängigkeit von den Charakteristika der teilnehmenden Personen, insbesondere dem Lebensalter, Körpergewicht und der Qualität der glykämischen Kontrolle.

Eine Metaanalyse von Studien über eine Zusatztherapie von Liraglutid bei Menschen mit Typ-1-Diabetes konnte eine moderate HbA_1c_-Reduktion, eine Reduktion der täglichen Insulindosis und eine deutliche Abnahme des Körpergewichts zeigen [58]. Der Einsatz von SGLT-2-Inhibitoren bei Menschen mit Typ-1-Diabetes ist aufgrund eines erhöhten Risikos für normoglykämische Ketoazidosen nur in ausgewählten Fällen bei Betreuung in einem Diabeteszentrum zu empfehlen [19]. Es sollte jedoch ein Einsatz von SGLT-2-Inhibitoren bei Herzinsuffizienz oder chronischer Nierenschädigung nach sorgfältiger Aufklärung hinsichtlich des Verhaltens in Sondersituationen (z. B. interkurrente Erkrankungen) erwogen werden, und, wenn erforderlich, sollte eine Kontrolle mittels Selbst-Ketonkörperbestimmung erfolgen.

### Weitere Autoimmunerkrankungen bei Typ-1-Diabetes

Entsprechend Literaturangaben entwickeln bis zu 30 % der Menschen mit Typ-1-Diabetes Autoimmunerkrankungen an weiteren Organsystemen [20, 60] Eine Autoimmunthyreoiditis (Morbus Hashimoto oder Basedow) tritt bei 15–30 % der Menschen mit Typ-1-Diabetes auf, eine Autoimmungastritis und/oder perniziöse Anämie bei 5–10 %, eine Zöliakie bei 4–9 %, ein Morbus Addison bei rund 0,5 % und eine Vitiligo bei 2–10 %. Auch das Risiko zur Entwicklung einer Autoimmunhepatitis und einer Myasthenia gravis ist erhöht [53]. Die Diagnostik beruht auf der serologischen Bestimmung organspezifischer Antikörper [60, 61].

Auswertungen von Daten aus dem Swedish National Diabetes Register bestätigen dies für Kinder und Jugendliche mit Typ-1-Diabetes: Über einen Beobachtungszeitraum von 19 Jahren seit Diagnose des Diabetes ist v. a. das Risiko zur Entwicklung einer atrophen Gastritis, eines Morbus Addison, einer Zöliakie, Schilddrüsenerkrankung und Autoimmunhepatitis gegenüber der nichtdiabetischen Population signifikant erhöht [62].

Das sog. *polyglanduläre Autoimmunsyndrom I und II* ist grundsätzlich mit einem erhöhten Risiko für die Manifestation eines Typ-1-Diabetes assoziiert, rund 20 % der betroffenen Personen entwickeln einen Typ-1-Diabetes [20, 63, 64]. Das Syndrom Typ I stellt eine seltene genetische Erkrankung dar, die auf eine Mutation des autoimmun-regulatorischen (AIRE) Gens zurückzuführen ist und ein autosomal-rezessives Vererbungsmuster aufweist [65]. Die Diagnose erfolgt bei Vorliegen von 2 oder mehreren Teilsymptomen, einschließlich einer mukokutanen Candidiasis, ektodermalen Dysplasien, einer Nebenniereninsuffizienz und/oder eines Hypoparathyreoidismus. Die charakteristischen Symptome sind häufig bereits im Kindesalter nachweisbar.

Dem polyglandulären Autoimmunsyndrom II liegt die Assoziation einer endokrinologischen Autoimmunerkrankung mit Einbeziehung von weiteren Organsystemen zugrunde. Charakteristika des Typ-I-Syndroms, insbesondere Mutationen des *AIRE*-Gens, sind nicht nachweisbar. Die Häufigkeit des polyglandulären Autoimmunsyndroms Typ II beträgt 1/20.000 mit einem Überwiegen von Frauen gegenüber Männern im Verhältnis von 3/1. Die höchste Inzidenzrate findet sich im Lebensalter zwischen 20 und 60 Jahren.

#### Autoimmunerkrankungen der Schilddrüse

Autoimmunerkrankungen der Schilddrüse führen zum klinischen Bild der Hashimoto-Thyreoiditis bzw. eines Morbus Basedow. Im Rahmen der Hashimoto-Thyreoiditis kommt es zum Auftreten von Antikörpern gegen die Thyreoperoxidase (TPO) oder Thyreoglobulin sowie zu einer Erhöhung der TSH-Konzentration. Thyreoperoxidase-Antikörper finden sich bei 15–30 % der Erwachsenen mit Typ-1-Diabetes. Die Prävalenz liegt damit deutlich höher als in der nichtdiabetischen Bevölkerung mit 2–10 % [66, 67]. Rund 50 % der TPO-Antikörper-positiven Menschen mit Typ-1-Diabetes zeigen einen Übergang in eine manifeste Schilddrüsenerkrankung. Eine subklinische Hypothyreose findet sich bei 13–20 % der Menschen mit Typ-1-Diabetes, eine subklinische Hyperthyreose bei 6–10 % gegenüber 0,1–2 % in der nichtdiabetischen Bevölkerung [67].

Unter Berücksichtigung dieser Datenlage wird in den Leitlinien von Fachgesellschaften wie der ADA eine regelmäßige Kontrolle des TSH-Wertes als Screening auf eine Schilddrüsenfunktionsstörung empfohlen [20, 53]. Die ISPAD empfiehlt, bei Kindern und Jugendlichen alle 2 Jahre den TSH-Wert und die Schilddrüsenantikörper zu bestimmen [68].

#### Zöliakie

Die Prävalenz der Zöliakie beträgt bei Menschen mit Typ-1-Diabetes zwischen 1 und 8 % gegenüber 0,5 % in der Allgemeinbevölkerung [20, 69, 70]. Bei Kindern wird ein Screening auf Zöliakie initial bei Diagnosestellung eines Typ-1-Diabetes mellitus empfohlen sowie regelmäßige weitere Kontrollen in einem 2‑ bis 5‑jährlichen Intervall [20, 71]. Bei Erwachsenen Menschen mit Typ-1-Diabetes mellitus sollte eine diagnostische Abklärung auf Zöliakie bei klinischer Symptomatik erfolgen [20].

#### Autoimmungastritis und Perniziosa

Die Häufigkeit der Autoimmungastritis ist bei Menschen mit Typ-1-Diabetes mit 5–10 % gegenüber der nichtdiabetischen Bevölkerung mit 2–4 % um das 3‑ bis 5‑Fache erhöht [71]. Das Krankheitsbild kann als Teilsymptom des polyglandulären Autoimmunsyndroms auftreten. Eine zumindest 1‑mal jährliche Bestimmung des Blutbilds und des Vitamin‑B_12_-Spiegels ist deshalb bei Patienten mit Typ-1-Diabetes empfehlenswert, um Folgekomplikationen zu vermeiden [20, 72].

#### Morbus Addison

Antikörper gegen die 21-Hydroxylase finden sich bei 0,7–3 % der Menschen mit Typ-1-Diabetes gegenüber maximal 0,6 % in der nichtdiabetischen Bevölkerung [73]. Entsprechend Literaturangaben beträgt die jährliche Inzidenz eines klinisch manifesten Morbus Addison 20 % [73]. Die klinischen Symptome des Morbus Addison wie Übelkeit, Erbrechen, Hypotonie, Gewichtsabnahme und Anorexie können beim manifesten Diabetes als Folge einer inadäquaten Behandlung bzw. Therapienebenwirkung missinterpretiert werden. Besondere Beachtung muss die erhöhte Neigung zu Hypoglykämien finden.

#### Vitiligo

Der Vitiligo liegt eine Autoimmunerkrankung mit der Ausbildung von Antikörpern gegenüber Melanozyten zugrunde. Bei Vitiligo ist das Risiko für die Manifestation von weiteren Autoimmunerkrankungen erhöht und beträgt rund 10 % für das Auftreten eines Typ-1-Diabetes mellitus [74].

#### Screeningempfehlungen

Unter Bezugnahme auf die Literatur und internationale Leitlinienempfehlungen:

- TSH-Kontrolle jedes 2. Jahr bei asymptomatischen und Antikörper-negativen Personen, ansonsten häufiger [20].
- Zöliakie-Screening bei Erstdiagnose eines Typ-1-Diabetes mellitus bei Kindern, in weiterer Folge alle 2 Jahre [71]. Berücksichtigt werden muss dabei das bei Menschen mit Typ-1-Diabetes erhöhte Risiko für einen IgA-Mangel mit falsch negativen serologischen Testergebnissen [75, 76].
- Screening auf Nebenniereninsuffizienz jedes 2. Jahr [77]. Kurzfristiger bei Auftreten eines klinischen Verdachts auf Morbus Addison. Bei positivem Nebennierenrinden-Antikörperbefund regelmäßige ACTH-Verlaufskontrolle und ggf. weitere Abklärung [20].

## Interessenkonflikt

M. Lechleitner: keine Interessenkonflikte. S. Kaser hat Vortragshonorare bzw. Beraterhonorare erhalten von Novo Nordisk, Eli Lilly, Sanofi. S. Hofer hat Vortragshonorare und Unterstützung bei Forschungsprojekten erhalten von Eli Lilly, Sanofi, Ypsomed, Insulet, Dexcom. F. Hoppichler hat Vortragshonorare erhalten von Boehringer Ingelheim, Eli Lilly, Sanofi, Novo Nordisk. M. Roden hat Vortragshonorare und Unterstützung bei Forschungsprojekten erhalten von AstraZeneca, Boehringer Ingelheim, Echosens, Eli Lilly, Intercept Pharma, Madrigal, MSD, Novo Nordisk, Sanofi, Synlab, Target RWE. R. Weitgasser hat Vortragshonorare und Unterstützung bei Kongressgebühren erhalten von Eli Lilly, Boehringer Ingelheim, Novo Nordisk, Abbott. L. Fruehwald hat Vortagshonorare und Unterstützung bei Kongressgebühren erhalten von Eli Lilly, Boehringer Ingelheim, Novo Nordisk, Abbott. B. Ludvik hat Vortragshonorare und Unterstützung bei Forschungsprojekten erhalten von Amgen, AstraZeneca, Boehringer Ingelheim, Daiichi Sankyo, Eli Lilly, Novo Nordisk. A. Feder hat Vortragshonorare und Unterstützung für Kongressgebühren erhalten von Eli Lilly. Y. Winhofer-Stöckl hat Vortragshonorare und Kongressunterstützung erhalten von Abbott, Dexcom, Eli Lilly, Novo Nordisk, Roche Diabetes Care, Sanofi Aventis. A. Kautzky-Willer hat Vortragshonorare und Unterstützung bei Forschungsprojekten erhalten von Eli Lilly, Novo Nordisk. G. Schernthaner: keine Interessenkonflikte. T. Wascher hat Vortragshonorare von Dexcom, Abbott, Eli Lilly und Novo Nordisk erhalten. M. Clodi hat Honorare für Vorträge, Studien und Beratung erhalten von Novo Nordisk, Eli Lilly, Boehringer Ingelheim, AstraZeneca, Novartis, Daiichi Sankyo, Amgen, Wörwag, Sanofi, Amarin, Roche, Bayer.
